# Effective Use of *Euphorbia milii* DCM Root Extract Encapsulated by Thermosensitive Immunoliposomes for Targeted Drug Delivery in Prostate Cancer Cells

**DOI:** 10.3390/cimb46110714

**Published:** 2024-10-27

**Authors:** Keamogetswe Riet, Ayodeji Adegoke, Samson Mashele, Mamello Sekhoacha

**Affiliations:** 1 Department of Health Sciences, Central University of Technology, Bloemfontein 9300, South Africa; 219015986@stud.cut.ac.za (K.R.); smashele@cut.ac.za (S.M.); 2 Department of Pharmacology, University of the Free State, Bloemfontein 9300, South Africa; adegoke.am@ufs.ac.za; 3 Cancer Research and Molecular Biology Laboratories, Department of Biochemistry, College of Medicine, University of Ibadan, Ibadan 200005, Nigeria

**Keywords:** docetaxel, liposomes, prostate–specific membrane, targeted drug delivery, thermosensitive, immunosensitive

## Abstract

The delivery of anticancer drugs using nanotechnology is a promising approach aimed at improving the therapeutic efficacy and reducing the toxicity of chemotherapeutic agents. Liposomes were prepared using HSPC: DSPE–PEG–2000: DSPE–PEG2000–maleimide in the ratio of 4:1:0.2 and conjugated with a PSA antibody. *Euphorbia milii* extract (EME), doxorubicin (Dox), and docetaxel (Doc) encapsulated in temperature–sensitive immunoliposomes were investigated for their activities against the prostate cancer LNCap and DU145 cell lines. Organic extracts of EME leaves, roots, and stems were screened against both cell lines, inhibiting more than 50% of cell culture at concentrations of 10 μg/mL. The immunoliposomes incorporating the EME and docetaxel were active against the LNCap cells when exposed to heat at 39–40 °C. The liposomes not exposed to heat were inactive against the LNCap cells. The developed heat-sensitive immunoliposomes used for the delivery of both the EME and chemotherapeutic agents was able to successfully release the entrapped contents upon heat exposure above the phase transition temperature of the liposome membrane. The heat-sensitive immunoliposomes conjugated with a PSA antibody encapsulated the extract successfully and showed better cell antiproliferation efficacy against the prostate cancer cell lines in the presence of heat.

## 1. Introduction

One in fifteen men in South Africa has a high chance of developing prostate cancer in their lifetime. African men have the highest reported incidence rates of prostate cancer in the world [1]. Prostate cancer is projected to exponentially grow globally to 1.7 million new cases and approximately 499,000 deaths by 2030 [2]. Treatments such as chemotherapy, surgery, hormone, and radiation therapy have been successful for the treatment of different cancers, including prostate cancer. The significant challenges facing current prostate cancer treatment are the side effects, which include killing normal cells and causing erectile dysfunction [3]. Nanotechnology has attracted much interest over the years regarding drug delivery because of its unique properties that increase the drug efficacy and selectivity and reduce the cytotoxicity [4]. The use of nanotechnology in the treatment of cancer appears to be a promising alternative with reduced side effects [5]. A promising type of nanoparticle is a liposome, which is a small, closed lipid vesicle comprising a phospholipid bilayer containing the aqueous center [6]. The stability and integrity of liposomes rely on their chemical composition. The ability of liposomes to entrap hydrophilic and lipophilic elements allows for the encapsulation of a wide range of drugs. The hydrophilic molecules are entrapped in the aqueous center, and hydrophobic molecules are in the lipid bilayer. When compared with other nanoparticles, such as gold, polymeric, silica, and iron oxide nanoparticles, liposomes have many advantages, despite their high current costs and the possibility of instability. Liposomes are effective drug carriers since they are noncovalent aggregates, and they can be used as delivery vehicles that transport drugs to the cancerous site. Their electric charge, lipid composition, and size can be easily modulated. The concentration of a drug encapsulated in liposomes can be determined by using various techniques, such as UV–vis detection and HPLC, depending on the active compound inside the liposomes [7,8]. Liposomes are easily metabolized and biodegradable in vivo. They can entrap various solutes with different characteristics and molecular weights; be surface–modified by hydrophilic polymers, such as polyethylene glycol (PEG); and avoid renal clearance and rapid uptake by cells of the reticuloendothelial system (RES), and thereby maximize the blood circulation time [9]. Liposomes can be sensitive and responsive to stimuli, such as pH, light, redox, and temperature [10]. This study explored the use of liposomes that were sensitive to temperature. Thermosensitive liposomes release the drug when exposed to heat at 39–40 °C [11]. Heat-sensitive liposomes enable effective drug delivery to a targeted site because heat increases the vascular permeability, interstitial transport, and precision of the time the drug is released from the liposome into the tumor. Temperature–sensitive liposomes have an extended circulation time, which enables them to passively release the drug so that it accumulates at the desired site [12]. These advantages made it suitable to be used as a delivery vehicle to meet the objectives of this study. The overexpression of cell receptors on the outer surface of cancer cells in tumors facilitates cancer cell recognition by targeting moieties conjugated on drug delivery systems. Anticancer therapy can target ligands associated with tumors, such as antibody nucleic acids, peptides, oligosaccharides, carbohydrates, and vitamins, which can be conjugated on the surface of liposomes [13,14]. After the targeted liposomes offload into the tumor interstitial space, ligand–receptor interactions increase the cellular internalization (for example, interactions between antigens and antibodies). Compared with other ligands, antibody–conjugated liposomes, which are also termed immunoliposomes, have attracted considerable attention as a targeted therapy [15]. Prostate cancer has the prostate–specific membrane antigen (PSMA), which is found in different prostatic tissue. The PSMA improves the therapeutic outcomes of nanoparticles in prostate cancer treatment [4].

To overcome treatment challenges, the development of target–specific and controlled drug release nano–delivery systems was explored. The current study aimed to overcome the challenges of the non-specific and uncontrolled drug delivery faced by chemotherapy by exploring the cancer cell growth inhibitory effect of a selected medicinal plant and the development of a controlled, target–specific nano–delivery system for prostate cancer. This study was conducted with liposomes encapsulating an active *E. milii* plant extract and standard prostate cancer drugs, namely, doxorubicin and docetaxel, for delivery into selected prostate cancer cell lines. Medicinal plants are seen as an alternative to chemotherapeutic drugs, as they contain a wide variety of bioactive compounds that possess anticancer, antibacterial, and antimicrobial properties. Secondary metabolites derived from medicinal plants have been the primary source of medical drug discoveries. Drugs such as vinblastine, paclitaxel, vincristine, etoposide, and taxel are examples of anticancer drugs derived from plants [16]. The use of *E. milii* in this study stemmed from previous reports about the anticancer potential of the plant and very limited studies on its use against prostate cancer. In general, *E. milii* is well known for its use in cancer therapy [17,18]. *E. milii* is commonly known as the ‘Christ of Thorns’; it is a woody, spiny succulent plant that can grow up to 1.8 m high, the spines can grow up to 3 cm, and it has green leaves and flowers ranging through colors like red, pink, white, and yellow. The plant belongs to the euphorbia genus, which is commonly found in Pakistan, China, and Madagascar. *E. milii* grows in bush and forest habitats but usually in rocky areas. The plant is native to southern and western Madagascar and is found at elevations between 20 and 1609 m above sea level. The plant is also found in other countries, like Brazil and Pakistan [19]. *E. milii* is used to treat warts; hay fever; digestive problems; and breathing disorders, like asthma and bronchitis [17]. In Brazil, *E. milii’s* latex is used to treat warts. In other countries, it is commonly used in cancer treatment. The bulbs are widely used to treat many illnesses, such as indigestion, stomach aches, constipation, diarrhea, and nausea. *E. milii* is known to have anticancer, antidiabetic, and antimicrobial properties. These properties are attributed to different phytochemicals present in the plant [20]. Phytochemical studies of *E. milii* showed that flavonoids, triterpenes, saponins, phenols, and tannins can be found in various parts of the plant [18]. A lot of research on this plant was conducted with ethanol extract, but not with hexane, DCM, or methanol. Our current study provided new information on other extract solvents of *E. milii* [21,22].

## 2. Materials and Methods

### 2.1. Materials

The EM plant was collected from Gariep Nursery, 309 Cliffendale Dr., Faerie Glen, Pretoria 0081, South Africa, on 11 November 2020 at 11:00 (SAST) and identified by a taxonomist Dr. Pienaar, Department of Botany, University of the Free State, Bloemfontein, South Africa. All solvents, namely, hexane, dichloromethane, and methanol, used in this research, as well as the chemicals used for the phytochemical analysis, were purchased from Thermo Fisher Scientific, Johannesburg, South Africa. Human prostate carcinoma cells LNCap and DU145 cell were purchased from Cellonex. The DMEM: F12 and DMEM medium, fetal bovine serum (FBS), MTT assay, HSPC: DSPE–PEG–2000: DSPE–PEG2000–maleimide, docetaxel, doxorubicin, anti-PSMA antibody Clone: 107 1A4 (cat. no. SAB4200257), and all materials used to determine the encapsulation efficiency were purchased from Merck, Modderfontein, South Africa. The BCA protein assay (Pierce) was purchased from Thermo Fisher Scientific Johannesburg, South Africa. 

### 2.2. Plant Extraction

The plant was washed, cut into small pieces, left to dry, and then ground to a fine powder. Nine grams of the powder were suspended in 150 mL of hexane, dichloromethane, or methanol. The mixtures were placed in a rotary shaker for 48 h at 25 °C at a solid: solvent ratio of 1:16. Each mixture was then filtered using filter paper (Whatman® Maidstone). Filtrates were concentrated under a decreased pressure at 45 °C at 150 rpm using a rotary vacuum evaporator and transferred into a pre-weighed clean vial and shade–dried at room temperature; they were thereafter stored at 4 °C until required for use.

### 2.3. Phytochemical Analysis

A standard qualitative screening of the EM specimen was conducted to detect the presence of secondary metabolites. The plant powder was subjected to various phytochemical analysis methods to evaluate the presence of phytosterols, saponins, flavonoids, pentose, tannins, glycosides, alkaloids triterpenoids, and anthraquinones based on previously published protocols [23,24,25].

#### 2.3.1. Determination of Phytosterols

Chloroform (10 mL) was added to the powdered material (0.05 g), followed by the careful addition of concentrated H_2_SO_4_ (1 mL) down the test tube walls. The presence of a reddish–brown color in the chloroform layer was taken to be an indication that phytosterols were present. 

#### 2.3.2. Determination of Pentose

Distilled water (40 mL) was added to the powdered plant material (2 g), after which the mixture filtration was carried out. To a 2 mL filtrate portion, 2 mL of a hydrochloric acid and phloroglucinol mixture was added. Thereafter, the solution was warmed for 5 min. The formation of red coloration indicated the presence of pentose. 

#### 2.3.3. Determination of Tannins

Distilled water (20 mL) was added to the powdered plant material (0.5 g). Thereafter, the mixture was subjected to a high temperature by boiling, and the mixture was filtered before cooling. The treatment of the filtrate was carried out by the addition of three drops of ferric chloride (0.1%). The presence of tannins was confirmed by the formation of blue–black precipitates. 

#### 2.3.4. Determination of Glycosides

Acetic acid (2 mL) was added to the powdered plant material (0.5 g). This step was followed by treating the mixture with one drop of ferric chloride (0.1%) and the gentle addition of concentrated sulfuric acid (1 mL). The observation of a brown ring indicated that deoxy sugars were present.

#### 2.3.5. Determination of Triterpenoids

To 2 mg of powdered plant material, 1 mL of chloroform was added, and then 3 mL of concentrated sulfuric acid was added to the solution. A reddish–brown coloration interface indicated that triterpenoids were present. 

#### 2.3.6. Determination of Anthraquinones

A solution of 10% HCl (12 mL) was added to the powdered plant material (1 g). Thereafter, the solution was boiled for 5 min. It was filtered and allowed to cool before chloroform (10 mL) was added to the filtrate. A clean test tube was used to collect the chloroform layer, and a 10% ammonia (10 mL) solution was added to the chloroform mixture. The formation of a rose–pink top layer while shaking the mixture was taken to be an indication of the presence of anthraquinones. 

#### 2.3.7. Determination of Saponins

Distilled water (5 mL) was added to the powdered plant material (0.5 g), boiled, and then filtered. Distilled water (3 mL) was then poured into the resulting filtrate, and the solution was vortexed for five minutes. An indication of frosting confirmed the detection of saponins.

#### 2.3.8. Determination of Flavonoids

Ethyl acetate (10 mL) was added to the powdered plant material (0.5 g), and heat was applied to the resultant mixture for about three minutes and was thereafter cooled. The cooled mixture was filtered, and, to the filtrate (5 mL), a diluted solution of ammonia (1 mL) was added. The presence of flavonoids was confirmed by observing a yellow precipitate when the mixture was shaken. 

#### 2.3.9. Determination of Alkaloids

A solution of 1% HCl (2 mL) was added to the powdered plant material (0.2 g), and this step was followed by the addition of 1 mL to both the Meyer’s reagent and the Drangendorff’s reagent. The observed greenish/creamy precipitate (for Meyer’s reagent) and reddish–brown appearance (for Drangendorff’s reagent) were taken as indications of alkaloids being present. 

### 2.4. Cell Culture

The human prostate carcinoma LNCap and DU145 cells were cultured in the DMEM: F12 and DMEM medium, respectively; supplemented with a 10% fetal bovine serum (FBS); and incubated at 37 °C in 5% carbon dioxide until 80% confluence was reached. Following the methods by [26,27], the cell viability was determined using trypan blue staining with an automated cell counter (Cell Countess TM) to obtain a cell concentration of 1 × 10^5^ cells/mL per well in a 96-well plate, which was used in all the experiments. The cells were plated and incubated for 24 h to adhere. After 24 h of incubation, the medium was removed, and the cells were treated with 100 µL of 100, 10, and 1 μg/mL of extracts in triplicates.

### 2.5. Synthesis of Heat-Sensitive Liposomes

Liposomes were prepared and modified using the thin–film method. Briefly, in a round–bottom flask, liposomes that consisted of HSPC: DSPE–PEG–2000: DSPE–PEG2000–maleimide in the molar ratio of 4:1:0.2 (2 mg:0.5 mg:0.1 mg) were dissolved in 5 mL of chloroform, and 2 mg of EM DCM root extract/docetaxel were dissolved in 2 mL of methanol. The solutions were mixed, and the organic solvents were evaporated using a rotary evaporator at 60 °C for ~20 min to form a solid film and left overnight covered with parafilm. This process encapsulated the drugs inside the liposomes. The dry heat-sensitive nanoparticles were hydrated with 2 mL of PBS and vortexed for 10 s at 3200 rpm to suspend the lipid materials in the solution. The solution was allowed to stand at 4 °C overnight to efficiently hydrate the lipid materials [28].

### 2.6. Cytotoxic Activity of Docetaxel-Loaded Liposomes and EME Liposomes

To evaluate the cytotoxic activity of the temperature–sensitive immunoliposome encapsulation, the EM DCM root extract, as well as the prostate cancer standard drug (docetaxel), the cytotoxicity was measured using the MTT assay in the LNCap cell line. This measurement was carried out with and without exposure to heat. To enable intracellular bioavailability, heat was applied to trigger release from the liposomes after cellular uptake. To study the effect of drug release beyond the transition temperature of liposomes on the cell viability, a heating strategy was followed. The cells and formulations were incubated at 39–40 °C for 15 min, followed by incubation at 37 °C for 48 h. The control experiment included no application of heat. The cells were then treated with an MTT solution for 4 h. The formazan crystals that formed were solubilized using DMSO, and the absorbance was measured using a multimode plate reader at 540 nm [29].

### 2.7. Liposome Conjugation

Heat-sensitive liposomes that consisted of HSPC: DSPE–PEG2000 and DSPE–PEG2000–maleimide (DSPEPEG2000–Mal) at a ratio of 4:1:0.2 were prepared. The anti-PSMA antibody Clone: 107 1A4 (cat. no. SAB4200257) and liposomes were combined at a molar ratio of 1:10 (antibody/DSPE–PEG2000–Mal liposomes) for 4 h at 4 °C with constant stirring, and the anti-PSMA antibody was then conjugated to the liposomes by reacting sulfhydryl residues on the antibodies with the C–terminal maleimide groups of the PEG chains [29,30]. The solution was ultracentrifuged at 30,000× *g* rpm and 4 °C for 2 h to remove the unconjugated antibodies. The supernatant was collected to determine the amount of residual anti-PSMA antibodies using the BCA protein assay kit [28]. 

### 2.8. Weighing of Liposomes

The heat-sensitive liposomes that encapsulated the EM extract and liposomes that encapsulated the docetaxel were measured after they were successfully synthesized and conjugated by centrifuging the PBS–hydrated liposomes for 30 min at 90,000× *g* using an ultracentrifuge at 4 °C. Following the centrifugation, the supernatant and pellets were separated. The pellets were weighed in milligrams.

### 2.9. Protein Analysis

A BCA protein assay (Pierce) was used to quantify the amount of anti-PSMA antibody conjugation on the liposomes. The unconjugated antibody removal from the liposomes was undertaken using an ultracentrifuge for 1 h. After removing the unconjugated antibody, the supernatant was collected to evaluate the quantity of the anti-PSMA. A dilution scheme for the microplate procedure of the BCA kit was used. One ampule of 2 mg/mL albumin standard was used to produce a set of diluted standards. BSA standards were diluted to concentrations of 2000, 1500, 1000, 750, 500, 250, 125, and 25 μg/mL. The protein contents of the samples were thereafter determined using a standard curve obtained at 562 nm [30].

### 2.10. Characterization of Liposomes

#### 2.10.1. Transmission Electron Microscopy (TEM)

The liposomes were analyzed using negative stain electron microscopy with a FEI T20 transmission electron microscope, purchased from Field Electron and Ion, in Hillsboro Oregon, United States. About 5 μmol/mL of liposome suspension was applied to carbon–coated grids, and after 2 min, the surplus was drawn off with filter paper. A saturated uranyl acetate aqueous solution was used as a staining agent; the surplus was removed with distilled water. The morphology of the sample was analyzed using TEM at 80 kV [31].

#### 2.10.2. Scanning Electron Microscopy (SEM)

SEM was used to analyze the morphological uniformity (particle shape and size) of the liposomes. The shapes of the immunoliposomes were analyzed using the Tescan MIRA RISE SEM purchased from Tescan in Brno, Czech Republic. This SEM is equipped with a Gatan Imaging Filter, with a 15 eV window and a 794 slow–scan CCD camera that operated at 2.00 kV [31].

#### 2.10.3. Determination of Encapsulation Efficiency

To determine the amount of encapsulation, the liposomes were centrifuged for 30 min at 90,000× *g* using an ultracentrifuge at 4 °C. The pellets were washed 4 times with a 0.9% NaCI solution. Then, 2.75 mL of alcohol was added to a 0.25 mL liposome solution and incubated at 42 °C for 10 min to break down the heat-sensitive liposomes. After the incubation, the liposomes were left to cool. The free drugs were separated from the liposomes using centrifugation, and the supernatant that contained the free drugs was collected and adjusted with methanol for further analysis. The amount of drugs released in the supernatant was determined using HPLC. For doxorubicin and docetaxel, the HPLC analysis was performed using a C18 column with a mobile phase that contained acetonitrile and water (32:68, *v*/*v*) at a flow rate of 1.0 mL/min. The column temperature was kept at 25 °C, and the sample (10 µL) was injected. The peaks were monitored using fluorescence detection at a 480 nm excitation wavelength and a 550 nm emission wavelength using a fluorescence detector. For the EME, the HPLC analysis was performed using a C18 column with a mobile phase that contained acetonitrile and water (32:68, *v*/*v*) at a flow rate of 0.8 mL/min. The column temperature was kept at 25 °C, and the sample (10 µL) was injected. The peaks were monitored at a 230 nm excitation wavelength and a 280 nm emission wavelength using an HPLC fluorescence detector for 45 min.

### 2.11. Statistical Analysis

The results are expressed as the mean ± standard deviation. Data were analyzed using one–way analysis of variance (ANOVA). A post hoc (Bonferroni) test was used to calculate the IC50 values using GraphPad prism version 8 software. Excel was used to confirm the level of significance between means (*p*-values of less than 0.05 were considered significant). An inhibition percentage of ≥50% at concentrations ≤10 μg/mL was considered active.

## 3. Results

### 3.1. Phytochemical Composition of E. milii

Phytochemical analysis of the *E. milii* plant specimen was conducted to detect the presence of secondary metabolites. 

Table 1 shows that glycosides, triterpenoids, phytosterols, flavonoids, pentose, tannins, anthraquinones, saponins, and alkaloid phytochemicals were detected in the leaves, stems, and roots. Saponins and alkaloids were absent from the leaves and stems, triterpenoids were absent from the leaves and roots, and flavonoids were absent from the leaves. 

### 3.2. Growth Inhibition of DU145 Cell Anti-Proliferation Assay

**Figure 1:**
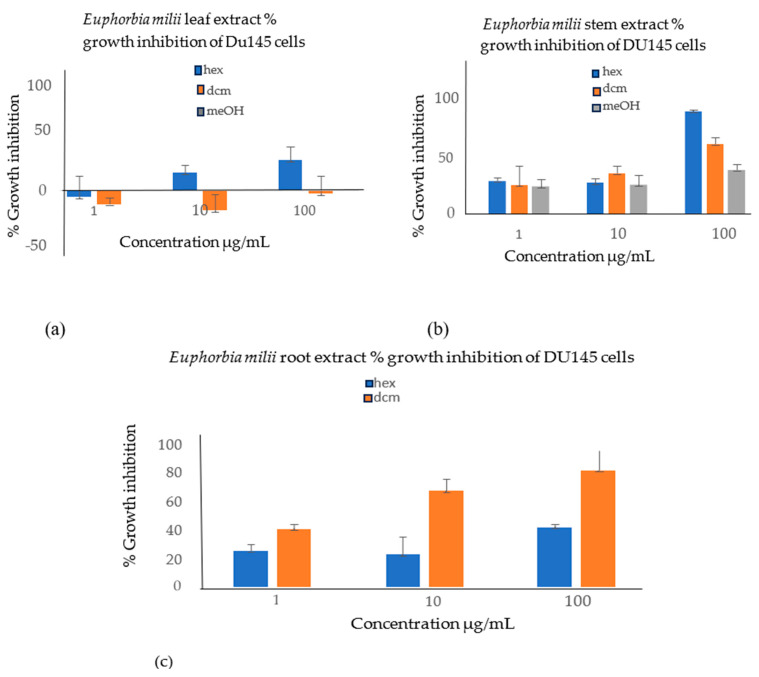
Cell growth inhibition of DU145 cells by different extracts (**a**) The hexane leaf extract showed less than 30% growth inhibition at all three concentrations. The DCM leaf extract showed no growth inhibition at all three concentrations. A significant difference (*p*-value < 0.05) was observed for the hexane leaf extract compared with the DCM leaf extract. (**b**) The DU145 cell growth inhibition by the EM hexane stem extract showed 114.2 ± 1.5% activity at 100 µg/mL but less than 50% activity at 10 µg/mL and 1 µg/mL. The DCM stem extract showed 37.4 ± 21.5% activity at 1 µg/mL, 49.7 ± 7.9% activity at 10 µg/mL, and 78 ± 1.8% activity at 100 µg/mL. No significant difference was observed for the hexane stem extract compared with the DCM stem extract (*p*-value < 0.36). Methanol extract was only obtained on the stems and not on the leaves and roots; hence, only the results for methanol stem extract are reported. The EM methanol stem extract showed no significant activity at all three concentrations. No significant difference was observed for the methanol stem extract when compared with both the hexane stem (*p*-value < 0.35) and DCM stem (*p*-value < 0.2) extracts. (**c**) The growth inhibition of the DU145 cells by the EM root extracts. The DCM root extract percentage inhibition of 50.1 ± 3.34% at 1 µg/mL, 67.51 ± 8.29% at 10 µg/mL, and 82.2 ± 18.41% at 100µg/mL. This one was the only extract that showed desirable activity when screened against the DU145 cells. A significant difference (*p*-value < 0.030) was observed for the hexane root extract compared with the DCM root extract.

**Figure 2:**
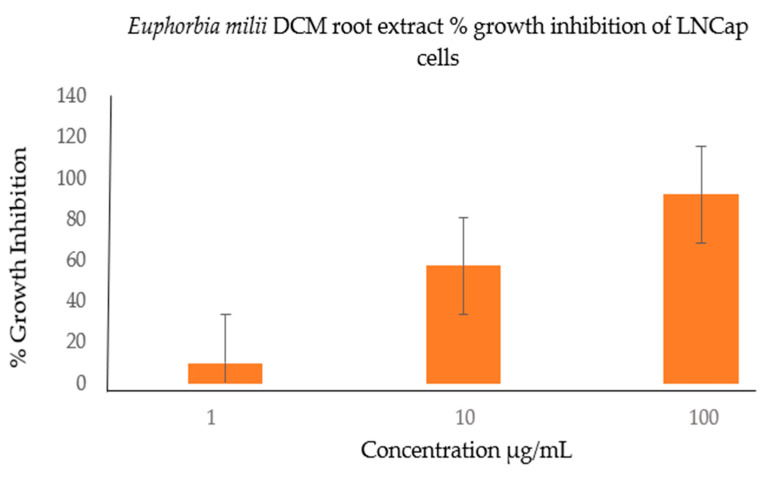
The growth inhibition of the LNCap cells when treated with different concentrations (100, 10, and 1 μg/mL) of the EM DCM root extract. The extract showed 91.98 ± 10.17% growth inhibition at 100 μg/mL and 51.7 ± 6.13% inhibition at 10 μg/mL. This extract showed significant activity against the LNCap cells, and, therefore, more dilutions of the extract were performed and screened for activity to obtain IC50 values, as seen in Figure 3 below.

**Figure 3:**
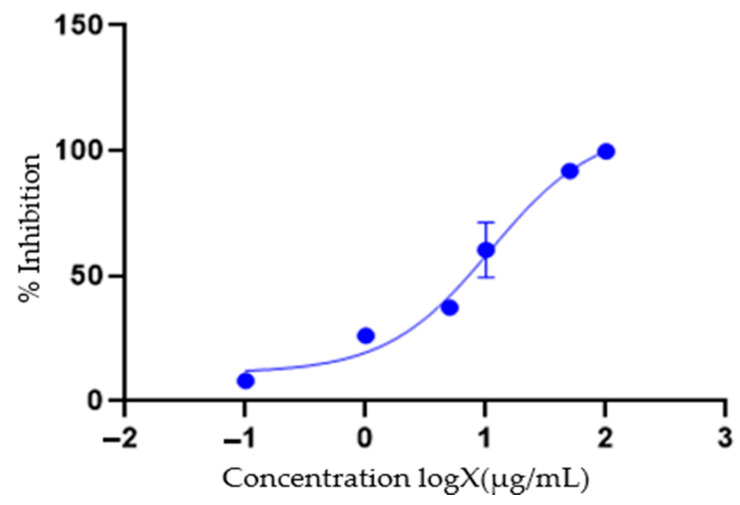
The cytotoxic activity of the *E. milii* DCM root extract on the LNCap cells. The results were analyzed with graphpad prism version 8 and the IC50 value was calculated to be 11.34 μg/mL.

**Figure 4:**
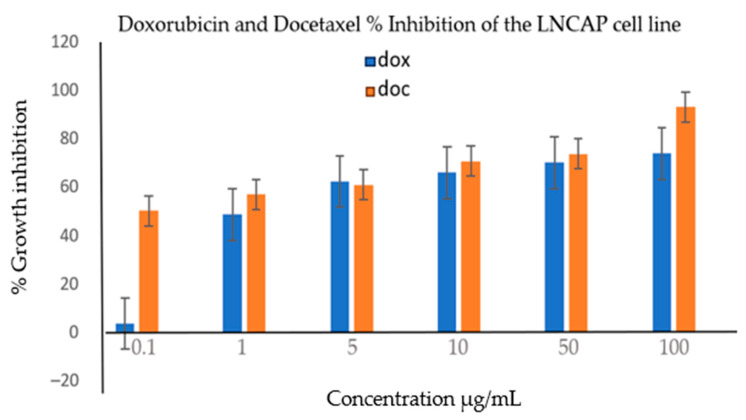
The cytotoxic activity of doxorubicin and docetaxel against the DU145 cell line. Doxorubicin (Dox) achieved ≥ 62.16 ± 2.5% inhibition activity at 5 µg/mL and above, with a maximum of more than 70% inhibition at 100 µg/mL. Docetaxel (Doc) was active, where it showed above 50% activity at all concentrations. No significant difference was observed for Dox when compared with Doc (*p*-value < 0.12).

**Figure 5:**
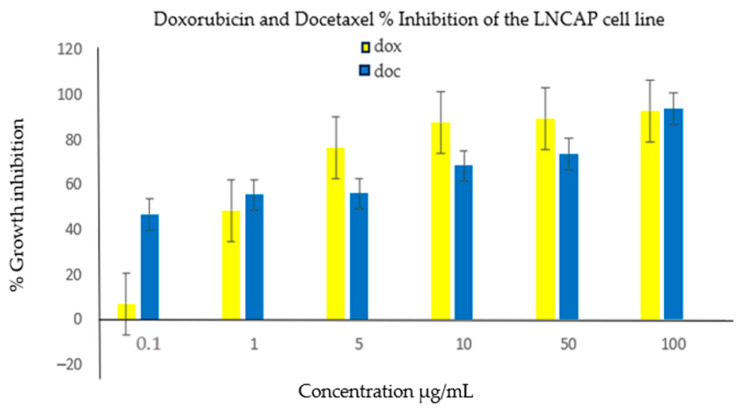
The cytotoxic activity of doxorubicin and docetaxel. Doxorubicin showed inhibition activities of 76.25 ± 7.05% at 5 µg/mL and more activity at higher concentrations, with 92.76 ± 3.2% at 100 µg/mL. Docetaxel showed an activity of 55.3 ± 3.66% inhibition at 1 µg/mL and more activity at higher concentrations, with 93.69 ± 2.41% at 100 µg/mL. No significant difference was observed for Dox when compared with Doc (*p*-value < 0.89).

The EM root, stem, and leaf extracts were screened for cytotoxic activity against the DU145 cells. Figure 1 shows the extracts tested in triplicate at 100 μg/mL, 10 μg/mL, and 1 μg/mL. The results are presented as the mean ± SD. The active extract was further tested against the LNCap cells, as seen in Figure 2 and Figure 3. Flavonoids, saponins, and glycosides are known to display cytotoxicity, anti-inflammatory and antioxidant properties, and thus are responsible for the anticancer activities in most plant extracts; these phytochemicals were detected in the active plant extract in this study (Figure 4 and Figure 5).

### 3.3. Characterization of Heat-Sensitive Liposomes

The heat-sensitive immunoliposomes were characterized using transmission electron microscopy (TEM) and scanning electron microscopy (SEM).

**Figure 6:**
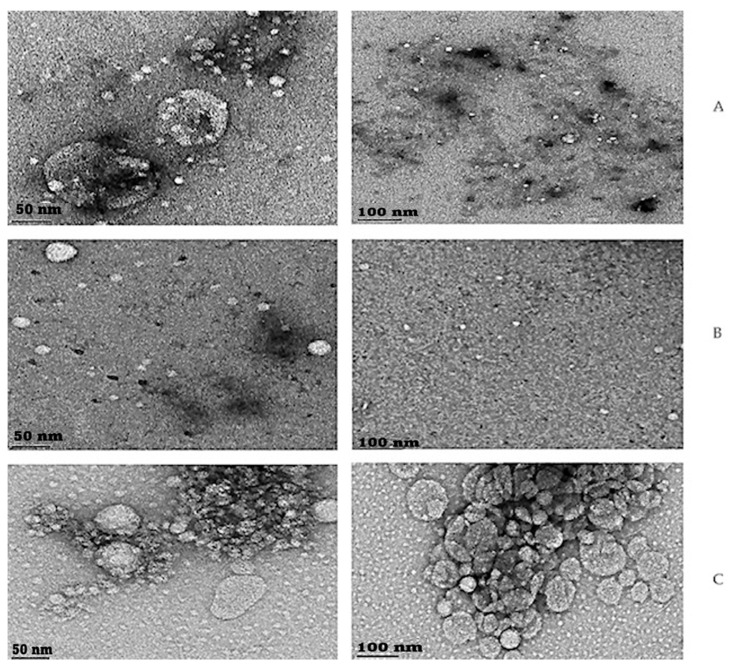
TEM images of immunoliposomes at a scale of 50 nm and 100 nm. (**A**) Transmission electron microscopy (TEM) was used to show the morphologies of the immunoliposomes at a scale of 100 and 50 nm. The TEM analysis of the empty liposomes was undertaken to confirm the formation of liposomes under experimental conditions. (**B**) TEM images of EME-loaded immunoliposomes at a scale of 50 and 100 nm. The TEM images show that the EME-loaded immunoliposomes were slightly bigger than the empty immunoliposomes at 50nm. In both the empty immunoliposomes and EME-loaded immunoliposomes, the TEM images indicate that the shape of the immunoliposomes appeared spherical and opaque; this shape indicates that the liposomes were stable and uniform in size. (**C**) TEM images of doxorubicin-loaded liposomes at a scale of 100 and 50 nm. The shape of the immunoliposomes appeared spherical and opaque. The larger circles that appear see through are marks caused by air-dried PBS.

**Figure 7:**
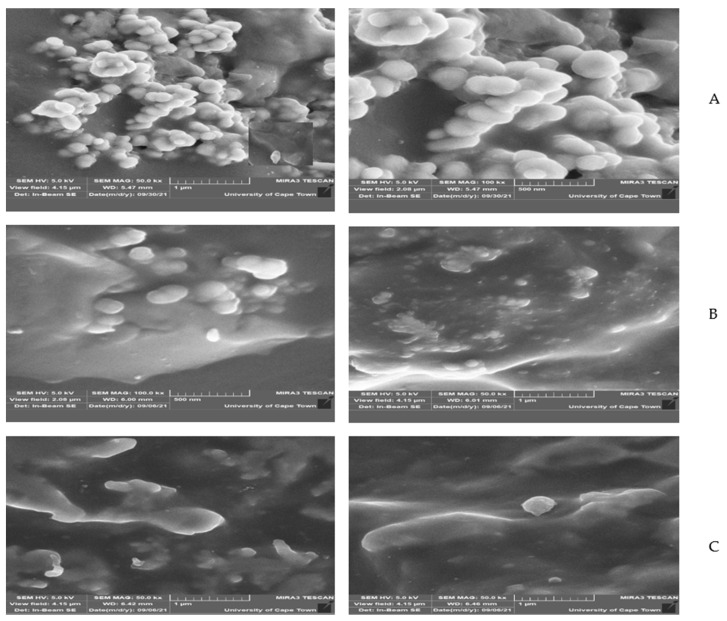
SEM images of the immunoliposomes. (**A**) SEM images of empty liposomes at a scale of 1 μm and 500 nm showing the morphologies of the synthesized heat-sensitive immunoliposomes. The highlighted insert shows a clear distinguishable morphology of the liposome. The impact the spherical morphology shows is that the liposomes are properly formed and are stable as the phospholipids assembled into bilayer membranes, which encloses an aqueous space successfully allowing the encapsulation of drugs. This morphology confirms that the liposomes can be used to successfully deliver chemotherapeutic agents in the desired concentration and retain their controlled release property. (**B**) SEM images of the EME-loaded liposomes at a scale of 500 nm and 1 µm. The morphologies of the liposomes appeared to be spherical at different parts of the grid. The liposomes were air-dried; therefore, the cloudy substance observed was air-dried PBS. (**C**) SEM images of the doxorubicin-loaded liposomes at a scale of 1 µm. The morphologies of the liposomes appeared spherical. The air-dried cloud-like appearance was background noise from the sampling grid.

Figure 6 and Figure 7 are TEM and SEM images respectively, showing the morphologies of the synthesized heat-sensitive immunoliposomes at a scale of 50 nm, 100 nm, 500 nm, and 1 μm. 

The TEM and SEM images of the empty liposomes show bigger shapes compared with the EM extract and drug–loaded liposomes. The encapsulation of the bioactive compounds in the bilayers of the lipids led to the liposomes’ size reduction.

### 3.4. Protein Conjugation

A BCA protein assay (Pierce) was used to quantify the amount of anti-PSMA antibody conjugation onto the liposomes. A calibration curve was plotted to determine the protein contents of the docetaxel–loaded immunoliposomes and EME-loaded immunoliposomes, where linear relationships were observed. The absorbances of the EME and standard drug samples at 526 nm were 1.14 and 1.774, respectively. The R–squared of the standard curve was 0.984. The concentrations of the protein in the *E. milii* and standard drug samples were calculated to be 990.1527 and 1287.712 µg/mL, respectively, as seen in Table 2, which indicates successful protein conjugation. 

### 3.5. HPLC/LCMS for Drug Encapsulation

The concentration of doxorubicin was calculated as 0.607 µg/mL, as seen in Table 3 below, and was therefore used as a reference to calculate the concentration of the EME using its peak area in relation to the doxorubicin pure drug peak area.

Table 4 shows the results for the drug–encapsulating thermosensitive liposome when run through LCMS and when the drugs were released by exposing the liposomes to heat at 40 °C. The EM mass 796 peak was investigated to quantify the amount of drug detected in the liposomes. The EM mass 796 peak area was larger than when the EME was not released. The EM mass 796 peak height was higher when the EME was released. The concentration of docetaxel detected when the drug was released was found to be 9 µg/mL, and when the liposomes were not exposed to 40 °C, the concentration was found to be 5.6 µg/mL. These results indicate that the doxorubicin, docetaxel, and *E. milii* extract were well encapsulated into the liposomes. 

The EME-encapsulating liposomes weighed 4.3 mg; this sample was divided into 2.15 mg for samples to be exposed to heat and 2.15 mg for samples that were not exposed to heat during the experiments. The docetaxel–encapsulating liposomes weighed 4.5 mg; this sample was divided into 2.25 mg for samples to be exposed to heat and 2.25 mg for samples that were not exposed to heat during the experiments. The docetaxel concentration inside the liposomes was calculated to be 839 μg/mL. Appendix A below show chromatograms of *E. milii,* doxorubicin and docetaxel.

### 3.6. Cytotoxic Activities of EME- and Docetaxel-Loaded Immunoliposomes

**Figure 8:**
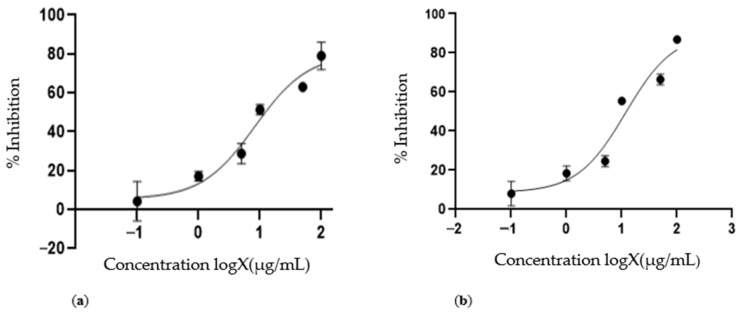
(**a**) Growth inhibition of LNCap cells by the EME-encapsulating heat-sensitive immunoliposomes when exposed to heat, where a 51.34 ± 13.0% inhibition was recorded at 10 μg/mL. The IC50 was calculated to be 8.44 μg/mL. (**b**) Growth inhibition of LNCap cells by docetaxel-encapsulating immunoligposomes when exposed to heat, where a 55.4 ± 4.8% inhibition was recorded at 10 μg/mL. The IC50 was calculated to be 11.74 μg/mL. The inhibition at 10 μg/mL showed that the docetaxel-encapsulating immunoliposomes had only a slightly higher inhibition than the EME. This trait showed that the EME was as cytotoxic to prostate cancer as docetaxel.

**Figure 9:**
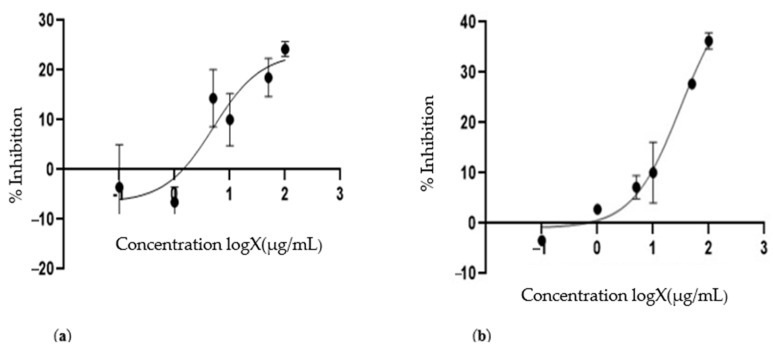
(**a**) Growth inhibition of LNCap by the EME-encapsulating immunoliposomes without exposure to heat. No significant activity was recorded at 10, 50, and 100 μg/mL. (**b**) The growth inhibition of LNCap cells by the docetaxel-encapsulating heat-sensitive immunoliposomes without exposure to heat. No significant activity was recorded at 10, 50, and 100 μg/mL. No significant cytotoxic inhibitory activity was recorded for the liposomes that encapsulated the extract or docetaxel without the presence of heat for all concentrations evaluated (activity less than 50% was termed insignificant). The above results show that upon the addition of a temperature that ranged from 39 to 40 °C, 51.34 ± 13.0% and 55.40 ± 4.8% cell inhibitions were recorded at 10 μg/mL for the liposomes that encapsulated the plant extract (Figure 8) and docetaxel (Figure 9), respectively. A significant difference (*p*-value < 0.0008) was observed for the EME-encapsulating heat-sensitive immunoliposomes when exposed to heat compared with no heat exposure. A significant difference (*p*-value < 0.00013) was observed for the docetaxel-encapsulating heat-sensitive immunoliposomes when exposed to heat compared with no heat exposure.

The cytotoxic activity of the temperature–sensitive immunoliposomes that encapsulated the EME and docetaxel was studied by measuring the cellular viability in the LNCap cells with and without exposure to heat. To ensure that the EME and docetaxel became intracellularly bioavailable, heat was applied to trigger release from the liposomes, as seen in Figure 8 and another experiment conducted without heat, illustrated in Figure 9. 

## 4. Discussion

This research, although still at a preliminary in vitro stage, represents a significant effort to reduce the mortality, poor treatment outcomes, and toxic side effects caused by currently used treatment options in prostate cancer patients. Due to the side effects and poor therapeutic outcomes of chemotherapy, targeted and controlled drug delivery has become a relevant technique for more effective and specific cancer treatment with reduced toxicity. To address these challenges, this study explored the use of heat-sensitive immunoliposomes that encapsulated either the EME or the standard prostate cancer drugs doxorubicin and docetaxel and assessed their cell growth inhibitory activities on selected prostate cancer cells. 

A phytochemical screening experiment was conducted to identify the various types of phytochemicals present in *E. milii*. The bioactive constituents obtainable from parts of plants are flavonoids, alkaloids, carotenoids, tannins, antioxidants, proteins, amino acids, and phenolic compounds [32]. As reported in Table 1, glycosides, triterpenoids, phytosterols, flavonoids, pentose, tannins, anthraquinones, saponins, and alkaloid phytochemicals were detected in the various parts of the plant examined, though saponins and flavonoids were not found in the leaves and stems, respectively. The cytotoxic activity results found in this study also show the inactivity of leaves and stems, which are in accordance with the findings of the absence of saponins and flavonoids, which were present in the roots of this plant. Saponins are glycosides that possess pharmacological properties, where the structural diversity of saponins has been linked to their anticancer activities [33]. Flavonoids play a role in various anticancer mechanisms, such as triggering apoptosis [34]. The cytotoxic activity of *E. milii* roots may be attributed to the presence of flavonoids and saponins. The findings from our study agree with those previously reported for the various parts of the plant analyzed [18,35,36]. Previously conducted research detailed that phytochemicals, such as tannins and flavonoids, can be biologically active [37,38]. Flavonoids, saponins, and glycosides are known to display cytotoxic, anti-inflammatory and antioxidant properties, and thus are responsible for the anticancer activities in most plant extracts [39]. The EM root, stem, and leaf extracts were initially screened for cytotoxic activity against the DU145 cell line; the results showed that the DCM root extract had a more significant cytotoxic effect against this cell line. A further analysis was performed against LNCap cells, where an IC50 of 11.34 µg/mL was obtained. Our study results are comparable with the results obtained by another group of researchers who examined EM against cervical cancer cells, where an IC50 of 22.1 ± 0.8 µg/mL was recorded [38]. Radi et al. found an IC50 of 10.41 µg/mL when the EM was screened against a breast cancer cell line [40]. The results obtained are significantly different from the results obtained by a study conducted on liver cancer cells showed an IC50 of 87.1 ± 9.4 µg/mL [41]. The anticancer activity obtained in our study could have been due to the flavonoids and saponins detected in the root extract. The EM root has been reported to contain more flavonoids and saponins than other parts of the plant [35], and this observation correlates with the results obtained from the phytochemical analysis performed in this study (Table 1), where saponins were only present in the root extract. Doxorubicin and docetaxel were used as positive controls and screened against the DU145 and LNCap prostate cancer cell lines. docetaxel showed a >50% cytotoxic activity against both the LNCap and DU145 cells at concentrations as low as 1 µg/mL, while doxorubicin only achieved more than 50% cytotoxicity at 5 µg/mL for both cell lines (Figure 4 and Figure 5). For the DU145 cells, no significant difference was observed for Dox when compared with Doc (*p*-value < 0.12). The growth inhibition activities observed for the positive controls were higher than those obtained previously [42], which showed that docetaxel had less than 50% cytotoxicity at a much higher concentration of 10 µg/mL. For the LNCap cells, no significant difference in the results was observed for Dox when compared with Doc (*p*-value < 0.89).

Nano–delivery systems, such as liposomes, were proven to improve the bioactivity of pharmacologically active compounds; this research informed the basis of this study, which was to prepare a liposomal delivery system that encapsulated the plant extract and standard prostate cancer drugs. Liposomes were prepared using HSPC:DSPE–PEG–2000:DSPE–PEG2000–maleimide in the molar ratio of 4:1:0.2 and were conjugated with a PSA antibody. A DSPE–PEG (2000)–amine was previously used for the synthesis of thermosensitive liposomal nanoparticles, mainly for the delivery of anticancer drugs and the synthesis of fluorescein isothiocyanate–loaded mesoporous silica nanoparticles for imaging applications [28]. It can be used with different functional molecules, such as DSPE–2000–maleimide, to prepare antibody–conjugated nanoparticles for improved cellular and receptor targeting [11]. After the preparation of the liposomes, the bicinchoninic acid (BCA) protein assay (Pierce) was used to quantify the amount of anti-PSMA antibodies conjugated onto the liposomes. The BCA assay utilizes peptide bonds in proteins to reduce Cu^2+^ to Cu^+^ at a rate that corresponds to the total protein in the sample. The bicinchoninic acid reagent forms a complex by binding with the Cu^+^. This complex then absorbs light at a 562 nm wavelength, which allows for a link between the sample’s protein concentration and absorbance [43,44]. The absorbances of the *E. milii* and docetaxel samples at 526 nm were 1.14 and 1.774, respectively. Using the equation y = mx + c, the protein concentrations of the liposomes that encapsulated the *E. milii* and standard drug samples were calculated to be 990.1527 and 1287.712 µg/mL, respectively. This calculation indicates successful conjugation of the anti-PSMA to the heat-sensitive liposomes for both samples.

To confirm the encapsulation percentage of the plant extract and docetaxel drug, HPLC and LCMS techniques were employed. Different masses of the *E. milii* extract were identified, as seen in Table 3. The mass 796 peak was prominent and therefore used in reference to the standard drug. Other masses were badly resolved and eluted as multiple peaks. Two 0.5 mg samples were analyzed using LCMS: sample 1, the *E. milii* extract, and sample 2, the doxorubicin (used as a control). A calibration curve was constructed with the doxorubicin standard and was 0.6 × 100 = 60 µg/mL, after considering the area of the peak of the *E. milii* extract in relation to the doxorubicin pure drug. The *E. milii* mass 796 and doxorubicin peak areas were 9.67 × 10^6^ and 6.78 × 10^6^, respectively. Therefore, the *E. milii* extract area was 2.89 times larger than that of the doxorubicin. The *E. milii* extract and doxorubicin peak heights were 6.87 × 10^5^ and 6.66 × 10^5^, respectively. The *E. milii* extract peak height was 0.21 times higher than that of doxorubicin. This difference indicates that more *E. milii* extract than doxorubicin was encapsulated in the liposomes. A chromatogram was constructed with the docetaxel standard drug, and the docetaxel concentration (100–fold diluted) was determined; it was found to be 839 μg/mL in a 2 mg sample. The plant extract concentration within a sample cannot be quantified because extracts are not pure compounds. Therefore, in this experiment, it was crucial to compare the plant extract chromatographic peaks to those obtained from a known drug sample. The results indicate that there was successful encapsulation of the *E. milii* extract, doxorubicin, and docetaxel.

Transmission and scanning electron microscopy were then used to further investigate the stability and morphology of the synthesized immunoliposomes. Both the TEM and SEM images indicated that the morphology of the immunoliposomes appeared spherical and opaque, which indicates that uniform stable liposomal delivery systems were formed. Furthermore, it is clear from the SEM and TEM images that the empty liposomes (Figure 6a and Figure 7a for the TEM and SEM, respectively) appeared bigger compared with the EME—(Figure 6b and Figure 7b for the TEM and SEM, respectively) and drug–loaded liposomes (Figure 6c and Figure 7c for the TEM and SEM, respectively). The encapsulation of the bioactive compounds in the bilayers of the lipids led to the liposomes’ size reduction, due to osmotic pressure on the liposomes, which led to the loss of aqueous molecules from the liposomal core [44]. This reduction in size is beneficial for the enhancement of bioavailability and improved drug delivery [45,46].

After the successful characterization of the liposomes, cell viability studies were performed. No significant cytotoxic inhibitory activity was recorded for the liposomes that encapsulated the extract or docetaxel without heat exposure for all the concentrations evaluated (activity less than 50% was termed insignificant). However, upon heat stimulation with a temperature that ranged from 39 to 40 °C, 51.34 ± 13.0% and 55.40 ± 4.8% cell inhibitions were recorded at 10 μg/mL for the liposomes that encapsulated the plant extract (Figure 8a) and docetaxel drug (Figure 8b), respectively. The IC50s were calculated to be 8.44 μg/mL and 11.74 μg/mL for the liposomes that encapsulated the plant extract and docetaxel drug, respectively. The HSPC in the constituents of the liposomal delivery systems may have been responsible for the significant activity recorded upon heat exposure. According to previous studies, HSPC was used to give temperature–sensitive liposomes their stability without the presence of heat, and upon heating to 40 °C, the structure of the liposomes was denatured. This step then led to the release of the bioactive compounds encapsulated in the liposomes, which provided cytotoxic activity [47,48]. 

## 5. Conclusions

Conventional forms of chemotherapeutic agents continue to experience drawbacks in the treatment of prostate cancer. There are various alternatives considered to remedy the present situation, among which is the use of alternative sources of pharmacologically active agents of plant origin, as well as the use of nanoparticles for the delivery of these chemotherapeutic agents. Liposomes are the most widely studied nano-delivery systems, which were shown to be a promising treatment alternative to the conventional delivery systems against cancer. This study focused on the development of a heat-sensitive immunoliposome nano-drug delivery system for the effective delivery of chemotherapeutic agents and *E. milii* plant extracts against prostate cancer cells. The *E. milii* DCM root extract showed a more significant cytotoxic effect against the tested prostate cancer cell lines (DU145 and LNCap). This study’s results confirm that the developed heat-sensitive immunoliposomes used for the delivery of both the *E. milii* plant extract and chemotherapeutic agents was able to successfully release the entrapped contents upon heat exposure above the phase transition temperature of the liposome membrane. The prepared, heat-sensitive immunoliposomes conjugated the active extract and showed better anticancer efficacy in vitro against the prostate cancer cell lines in the presence of heat than in the absence of heat. This heat-sensitive delivery system has controlled and targeted release properties that address the challenges faced by current prostate cancer treatment and has the potential for further analysis and development, which could potentiate its use as a safe and efficient delivery system for anticancer drugs against prostate cancer. It also provides revolutionary research alternatives for the treatment of various cancers, as well as possible use in other disciplines, such as gene therapy, which has the potential to develop specific and controlled personalized cancer treatment. The use of target–specific nano-delivery systems in personalized medicine will enable personalized treatment tailored to each cancer, which addresses currently faced treatment challenges, such as low bioavailability, uncontrolled drug release, the degradation of drugs, and non-specificity. However, it is crucial to emphasize the need to carry out further experiments, the lack of which were limitations to this study; in particular, further experiments include examining the release profile of the encapsulated drug over time and the characterization of liposomes, such as using DLS analysis to determine the average size of the liposomes, polydispersity index, and surface charge. 

## Figures and Tables

**Table 1:** Summary of phytochemical screening results. Legend: ‘+’, present; ‘−’, not detected.

Phytochemicals	Leaves	Stems	Roots
Glycosides	+	+	+
Phytosterols	+	+	+
Saponins	−	−	+
Anthraquinones	+	+	+
Pentose	+	+	+
Alkaloids	−	−	+
Tannins	+	+	+
Triterpenoids	−	+	−
Flavonoids	−	+	+

**Table 2:** The total protein contents of the EME and standard drug encapsulated by heat-sensitive immunoliposomes.

Samples	Concentration (µg/mL)
EME 1.41	Y = 817.47(1.41) − 162.48 = 990.1527
Standard drug 1.774	Y = 817.47(1.774) − 162.48 = 1287.712

**Table 3:** The integrated peak area and peak height values of the chromatograms for the selected masses in the EM extract sample (sample 1) and the concentration of doxorubicin (sample 2).

Sample Name	Analyte Peak Name	Analyte Peak Area (Counts)	Analyte Peak Height (cps)	Calculated Concentration (µg/mL)
EME	Mass 271	1.98 × 10^7^	1.23 × 10^6^	0
EME	Mass 301	1.66 × 10^7^	6.54 × 10^5^	0
EME	Mass 318	2.62 × 10^7^	1.44 × 10^6^	0
EME	Mass 352	4.73 × 10^6^	2.12 × 10^5^	0
EME	Mass 637	5.39 × 10^5^	1.57 × 10^4^	0
EME	Mass 678	1.28 × 10^5^	2.89 × 10^3^	0
EME	Mass 754	2.17 × 10^6^	1.74 × 10^5^	0
EME	Mass 796	9.67 × 10^6^	6.87 × 10^5^	0
EME	Dox	6.78 × 10^6^	6.66 × 10^5^	0.607

**Table 4:** The integrated peak area and height values of the chromatograms for the mass 796 analytes in the EME sample and the concentration of docetaxel in the docetaxel–encapsulating liposomes when the drugs were released and when they were not released from the liposomes.

Table Sample Name	Analyte Peak Name	Analyte Peak Area (Counts)	Analyte Peak Height (cps)	Calculated Concentration
Free EME	Mass 796	4.33 × 10^7^	2.24 × 10^6^	0
EME—encapsulating liposomes	Mass 796	1.69 × 10^7^	1.10 × 10^6^	0
Free DOC	DOC	1.75 × 10^6^	1.12 × 10^5^	9
DOC—encapsulating liposomes	DOC	1.1 × 10^6^	7.70 × 10^4^	5.6

Free EME refers to the EME released from the liposomes when exposed to heat. Free Doc refers to the docetaxel released from the liposomes when exposed to heat.

**Figure A1:**
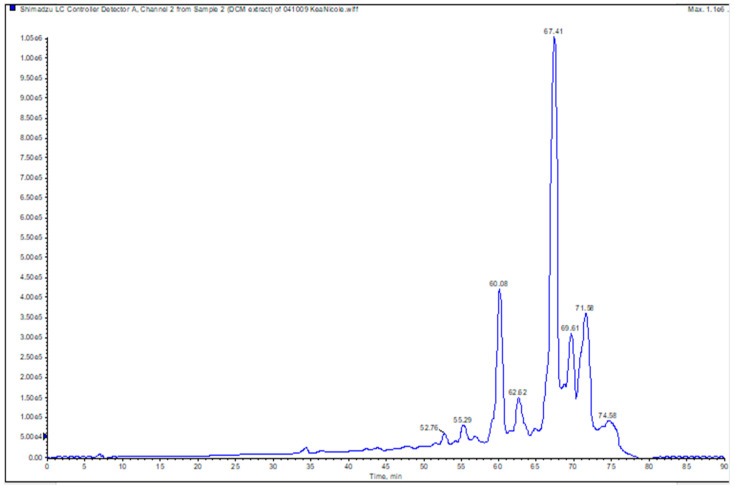
LCMS spectrum of the EME sample.

**Figure A2:**
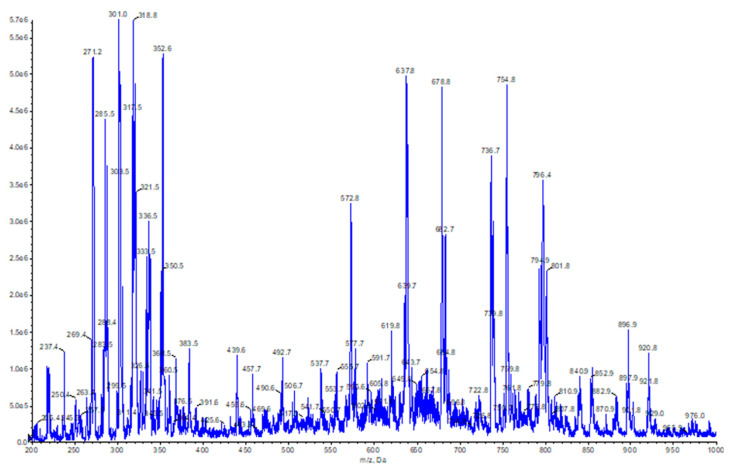
Several masses that were identified in the EM plant extract.

**Figure A3:**
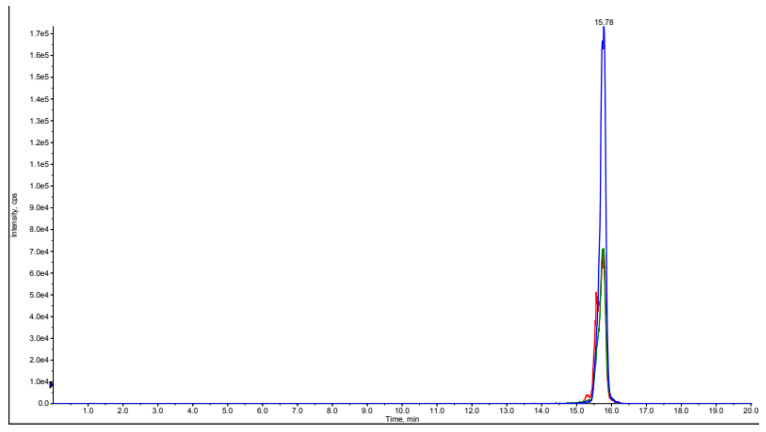
Chromatogram of mass 754 of the EME.

**Figure A4:**
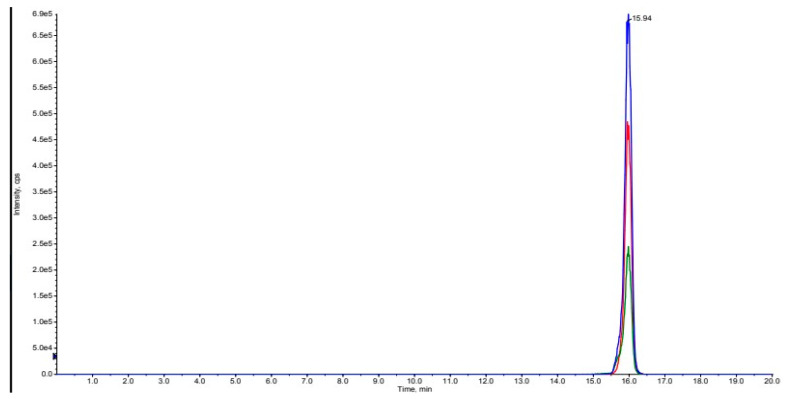
Chromatogram of mass 769 of the EME.

**Figure A5:**
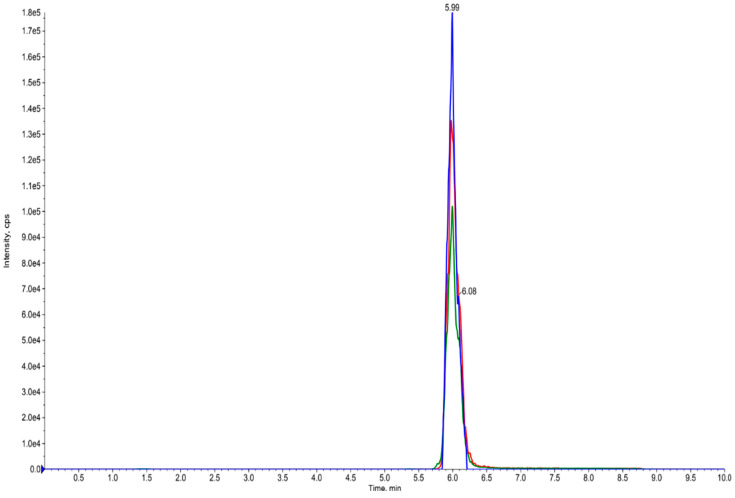
Chromatogram of docetaxel.

**Figure A6:**
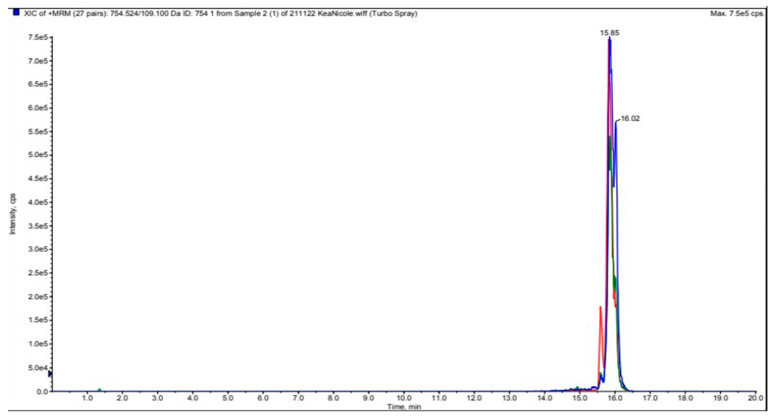
Chromatogram of mass 754 of the Euphorbia released.

**Figure A7:**
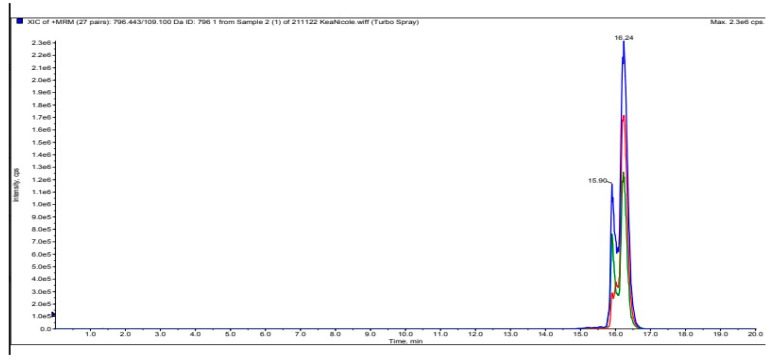
Chromatogram of mass 796 of the Euphorbia released.

**Figure A8:**
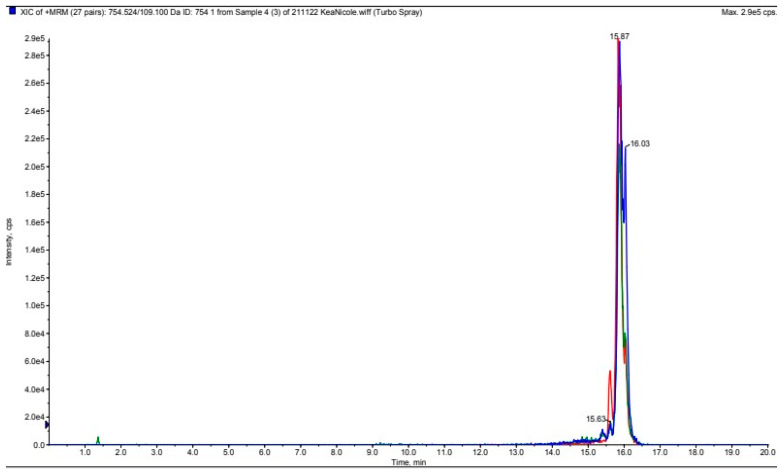
Chromatogram of mass 754 of the Euphorbia unreleased.

**Figure A9:**
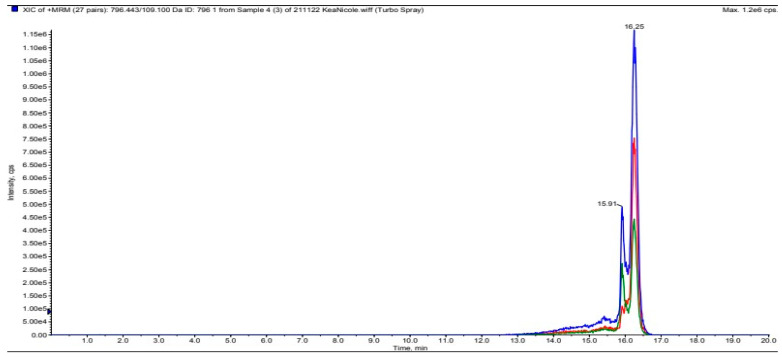
Chromatogram of mass 796 of unreleased Euphorbia.

**Figure A10:**
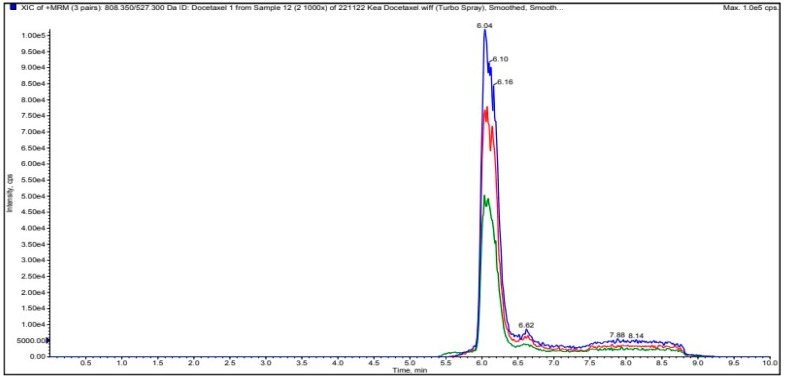
Chromatogram of released docetaxel.

**Figure A11:**
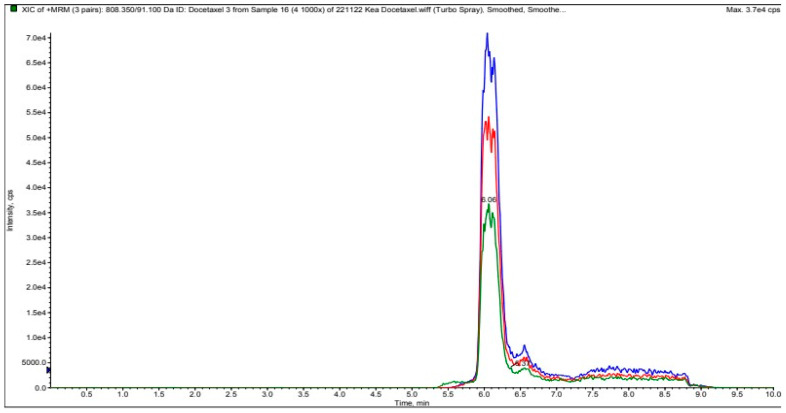
Chromatogram of unreleased docetaxel.

## Data Availability

The original contributions presented in this study are included in the article. There are no Supplementary Material. Further inquiries can be directed to the corresponding author.

## Abbreviations

DCM	Dichloromethane
MeOH	Methanol
PSMA	Prostate–specific membrane antigen
TSL	Thermosensitive liposomes
DMSO4	Dimethyl sulfoxide
DMEM	Dulbecco’s modified Eagle’s medium
FBS	Fetal bovine serum
TC	Transition temperature
HSCP	L-α-phosphatidylcholine, hydrogenated (soybean)

## Appendix A

The most intense peak of the EM extract was observed at 67 min, and Figure A1 shows the spectrum of all the masses in that peak between 200 and 1000 Da (in positive ionization mode).

The masses and the peak at 67 min were chosen as markers for the HPLC. Several masses were identified in the most intense chromatographic peak (at 67 min). The masses selected were 271, 301, 318, 352, 637, 678, 754, and 796 and were used as marker masses. There were only two masses that were separated as well–structured peaks: 754 and 796. The other masses were badly resolved and eluted as multiple peaks and then integrated as one to give the values above.
